# Platelet Protease Activated Receptor 1 Is Involved in the Hemostatic Effect of 20(*S*)-Protopanaxadiol by Regulating Calcium Signaling

**DOI:** 10.3389/fphar.2020.549150

**Published:** 2020-09-18

**Authors:** He Zhang, Daian Pan, Xingquan Wu, Wenjie Su, Xiaolei Tang, Daqing Zhao, Liwei Sun, Bailin Song, Xueyuan Bai, Xiangyan Li

**Affiliations:** ^1^ Research Center of Traditional Chinese Medicine, The Affiliated Hospital to Changchun University of Chinese Medicine, Changchun, China; ^2^ Department of Tuina, The Affiliated Hospital to Changchun University of Chinese Medicine, Changchun, China; ^3^ College of Pharmacy, Changchun University of Chinese Medicine, Changchun, China; ^4^ Key Laboratory of Active Substances and Biological Mechanisms of Ginseng Efficacy, Ministry of Education, Jilin Provincial Key Laboratory of BioMacromolecules of Chinese Medicine, Jilin Ginseng Academy, Changchun University of Chinese Medicine, Changchun, China

**Keywords:** 20(*S*)-protopanaxadiol, hemostatic effect, platelet, protease-activated receptor 1, vorapaxar

## Abstract

*Panax notoginseng* (Burk.) F.H. Chen has long been used to stop bleeding for hundreds of years in China. At present, only dencichine, notoginsenoside Ft1, and 20(*S*)-protopanaxadiol (PPD) showed hemostatic effect. However, the molecular mechanism of PPD on the platelet aggragetion needs to be further investigated. The study aims to evaluate the hemostatic effect of PPD and reveal its interacting targets using a series of experiments. In this study, the bleeding time was measured in mouse tail amputation and liver scratch models to evaluate hemostatic effect of PPD. The routine blood and plasma coagulation parameters in NS, HC, and PPD (2, 4, and 8 mg/kg) groups were measured using a blood analyzer. Platelet aggregation rate and ATP release were analyzed by a platelet aggregometer. Subsequently, the degranulation marker CD62P and PAC-1, and the concentrations of cytosolic Ca^2+^ ([Ca^2+^]_i_), cAMP, cGMP, and PAC-1 expressions were also assessed. We found that PPD shorted the bleeding time on the mouse tail amputation and liver scratch models and mainly increased blood platelet count in the rats after subcutaneous injection for 4 h. Meanwhile, PPD decreased APTT, increased FIB content, and directly induced platelet aggregation *in vitro*. In the absence of Ca^2+^, PPD induced the increase of [Ca^2+^]_i_ and slightly increased the levels of CD62P and PAC-1. After the addition of 1 mM Ca^2+^, PPD treatment markedly promoted platelet activation by promoting ATP level, releasing CD62P and increasing PAC-1 binding in washed platelets. Excitingly, PPD-induced changes including platelet aggregation, decreased cAMP content, and the increases of CD62P and PAC-1 were significantly reversed by protease-activated receptor 1 (PAR-1) antagonist, vorapaxar, which showed similar function as thrombin. In addition, molecular docking analysis and ELISA assay demonstrated that PPD had a promising docking score with -6.6 kcal/mol and increased PAR-1 expression in human platelets, which indicated that PAR-1 is involved in PPD-induced platelet aggregation by regulating calcium signaling. Collectively, our study could provide the new insights of PPD as an essential hemostatic ingredient in *Panax notoginseng* for the treatment of hemorrhagic disease.

## Introduction

Hemostasis is a pivotal process that prevents blood loss after blood vessel injury. This process is tightly regulated and depends on an intricate series of events involving platelets, vascular components, and plasma coagulation factors (Ivanciu and Stalker, 2015). In these processes, platelet activity is associated with the initiation of coagulation. As a consequence of vessel wall damage, subendothelial matrixes (such as collagen, von Willebrand factor (vWF), fibronectin, etc.) are exposed to the flowing blood; circulating platelets adhere to the subendothelial surfaces (Corral et al., 2002). During this process, the platelets change its shape, release its granule contents, and gradually form platelet-platelet aggregation by adhering with each other (de Queiroz et al., 2017). Extracellular Ca^2+^ entry is a crucial step in the activation, the change of shape and granules release of platelets (Ghoshal and Bhattacharyya, 2014; Harper and Sage, 2017). Some receptors such as the glycoprotein (GP) IIb/IIIa (fibrinogen receptor), GPIb/IX/V complex (vWF, thrombin, and P-selectin receptors), GPVI (collagen receptor), P2Y_12_ (ADP receptor), and protease-activated receptors (thrombin receptor) are exposed and activated on platelet membrane during shape change (de Queiroz et al., 2017). At the same time, granules components of platelet release, including 5-hydroxytryptamine (5-HT), ADP, adenosine triphosphate (ATP), histamine, CD63, P-selectin, platelet factor 4 (PF4), and vWF, could bind to their relevant receptors to maintain and amplify the initial platelet response and stimulate more circulating platelets that are recruited to form the aggregation (Blair and Flaumenhaft, 2009; Smyth et al., 2009).

Aggregated platelets ultimately form the “platelet plugs” or “white thrombi” at the injured vessel wall (Becker et al., 2018). The platelets plug initially formed is relatively unstable in primary hemostasis. Coagulation cascade and polymerization of fibrin could prolong secondary hemostasis (Ghoshal and Bhattacharyya, 2014). Activated platelets expose negatively charged phospholipids, which facilitates the activation of coagulation cascade (e.g., FVIIa, FIXa, FXa, and FV) (Lippi et al., 2009). This process involves a series of calcium-dependent conversions of proenzymes; finally, prothrombin is converted to thrombin. Furthermore, thrombin induces platelet aggregation and converts the soluble plasma protein fibrinogen into insoluble fibrin form a stabilizing mesh surrounding the platelets plugs (Rau et al., 2007). These cascades result in the formation of “red thrombus” to stop the bleeding. However, some patients during surgery or accident lose massive blood cells and have to use the hemostatic drugs, even required blood transfusions if necessary. Therefore, it could offer significant advantages for the development of hemostatic agent with excellent efficiency of hemostatic activity through multiple pathways as mentioned above.

Natural products from traditional Chinese medicine have a good hemostatic effect and fewer side effects. *Panax notoginseng* (Burk.) F. H. Chen (*P. notoginseng*), as a traditional Chinese medicine, is the most famous hemostatic elixir with unique characteristics called “hemostasis without stasis” for hundreds of years in China (Yang, 2015). Compendium of Materia Medica (Ben Cao Gang Mu) and Yu Qiu Yao Jie record that *P. notoginseng* could stop bleeding, disperse stasis blood and relieve pain, as well as cure multiple blood diseases. In Chinese Pharmacopeia, *P. notoginseng* promotes blood circulation and removes blood stasis, hemostasis, detumescence, and pain, which is used to treat hemoptysis, hematemesis, epistaxis, hemafecia, metrorrhagia, and metrostaxis, wound, chest and abdomen stabbing pain, swelling and blood stasis pain from knocks and falls, and so on (Yang et al., 2018).

**Figure 1:**
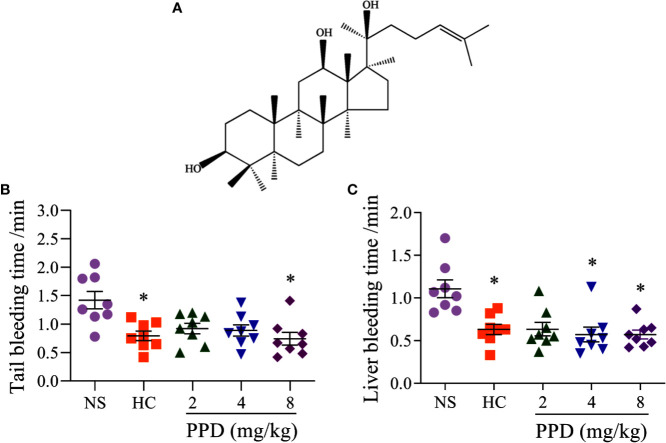
The effect of PPD on the mouse bleeding time. **(A)** The chemical structure of 20(S)-protopanaxadiol (PPD). **(B)** Tail bleeding time was measured in tail amputation model of mice treated with normal saline (NS), hemocoagulase (HC) or PPD (2, 4, and 8 mg/kg) for 4 h. **(C)** Liver bleeding time was measured in liver scratch model of mice treated with NS, HC, or PPD (2, 4, and 8 mg/kg) for 4 h. The data are expressed as mean ± SD (*n* = 8). *p < 0.05 compared to the NS group.

The characteristics of *P. notoginseng* in treating blood troubles have a bidirectional therapeutic effect both hemostatic and anti-thrombotic action (Wang et al., 2014). At present, there are a lot of studies about the ingredients of anti-thrombosis in *P. notoginseng*, for instance, protopanaxadiol-type ginsenoside Rb1 (He et al., 2007), Rg3 (Jeong et al., 2017), Rd and Rh2 (Gao et al., 2014), as well as protopanaxatriol-type ginsenoside Re (Hongyi et al., 2016), Rg1 (Zhou et al., 2014), Rg2 (Li et al., 2013), notoginsenoside Fc (Liu et al., 2018), and R1 (Yao et al., 2008), which can inhibit platelet aggregation and thrombus formation. However, there are few reports on hemostatic components from *P. notoginseng*. The dencichine (Huang et al., 2014; Ding et al., 2018) and notoginsenoside Ft1 (Gao et al., 2014; Liu et al., 2019) have shown that shorten the bleeding time of mice and promote the platelet aggregation, which have a significant hemostatic effect. Gao et al. reported that 20(S)-protopanaxadiol (PPD) increased ADP-induced platelet aggregation (Gao et al., 2014). PPD (**Figure 1A**), the aglycone of protopanaxadiol-type ginsenosides, is an important active component of *Panax notoginseng*(Burk.)F. H. Chen, *Panax ginseng* C. A. Mey or *Panax quinquefolium* L. Many studies have shown that protopanaxadiol-type ginsenoside Rb1, Rd, Rg3, Rh2, and compound K could metabolize to PPD by intestinal bacteria, acid, base, or enzymes (Akao et al., 1998; Shen et al., 2018). In recent years, PPD demonstrated a wide of pharmacological activities, such as anticancer (Sun et al., 2011; Ben-Eltriki et al., 2016), antidepressant (Xu et al., 2010), and vasorelaxant effect (Gan et al., 2016). However, the effect and molecular mechanism of PPD on platelet aggregation need to be clearly investigated in the platelet activation of hemostatic process. In this study, we investigated the function of PPD on platelet aggregation in different models and explored its molecular mechanism of hemostatic effect, which could provide an important insight on for the treatment of bleeding diseases.

## Materials and Methods

### Chemicals

20(S)-protopanaxadiol (PPD, purity of ≥98% by HPLC) was purchased from Shanghai yuanye Bio-Technology Co., Ltd (Shanghai, China). Hemocoagulase was purchased from Jinzhou Ahon Pharmaceutical Co, Ltd. (Liaoning, China). Prothrombin (PT), activated partial thromboplastin time (APTT), thrombin time (TT), and fibrinogen (FIB) kits were obtained from Nanjing Jiancheng Bioengineering Institute (Nanjing, China). Luciferin/luciferase reagent and thrombin were purchased from Chrono-Log Corporation (Pennsylvania, USA). Vorapaxar, ticagrelor, and seratrodast were purchased from MedChemExpress (New Jersey, USA). Cyclic adenosine monophosphate (cAMP), cyclic guanosine monophosphate (cGMP), and human protease activated receptor 1 ELISA kits were purchased from Sino Best Biological Technology Co., Ltd. (Shanghai, China). FITC-conjugated anti-human CD62P (P-selectin) and FITC-conjugated anti-human PAC-1 were purchased from BioLegend (California, USA). Fluo-3 AM calcium indicators and lactate dehydrogenase (LDH) cytotoxicity assay kits were purchased from Beyotime Biotechnology (Shanghai, China).

### Animals

Male Kunming mice (weighting, 20.0 ± 2.0 g) and male Wistar rats (weighting, 190.0 ± 10.0 g) were purchased from Liaoning Changsheng Biotechnology Co., Ltd [Animal license No. SCXK (Ji)-2016-0003]. The animals were housed under controlled temperature (25 ± 1°C), relative humidity (60 ± 5%) and a 12 h light/dark cycle with ad libitum access to food and water. All animal experiments were approved by the Bioethics Committee of Changchun University of Chinese Medicine and the Institutional Animal Care (approval no. 20190133), which was conducted based on the guideline for the use of laboratory animals.

### Bleeding Time Measurement

The measurements of bleeding time in mouse tail amputation and liver scratch models were established, according to previous methods with some modifications (Li et al., 2008; Gao et al., 2014; Yan et al., 2017). Briefly, 40 male Kunming mice (20.0 ± 2.0 g) were randomly divided into five groups, NS group (1% sodium carboxymethyl cellulose-normal saline, CMC-Na), HC group (1 KU/mL hemocoagulase), and 2, 4, and 8 mg/kg PPD groups. After the subcutaneous injection of drugs for 4 h, mice were anesthetized with 4% pentobarbital sodium *via* intraperitoneal injection (IP). In the tail amputation model, the tails of mice were transected with a sterile razor blade at the site that 10 mm apart from the tip and then immersed in 37°C normal saline. The bleeding time was defined as the time from the start of transection to bleeding cessation. Stop time of tail bleeding over 30 s was considered as bleeding time. In the liver scratch model, the liver injury was established by scratching the left lateral lobe with a 2-mL syringe about 1 cm to cause the liver to bleed. Then, the incision was dipped with filer paper at 10 s intervals until hemostasis. All of the mice were euthanized *via* cervical dislocation under anesthesia at the end of each experiment.

### Routine Blood Test and Plasma Coagulation Assay *In Vivo*

1% CMCNa-normal saline (NS group), 1 KU/mL hemocoagulase (HC group), and 2, 4, and 8 mg/kg PPD were subcutaneously injected into five groups of wistar male rats (*n* = 8), respectively. After 4 h treatment, rats were anesthetized with 4% pentobarbital sodium *via* IP to withdrawn blood samples from abdominal aorta and then placed in plastic tubes with EDTA and 3.8% sodium citrate, respectively. Blood samples with EDTA were detected routine blood by using XT-2000i automated hematology analyzer (Sysmex Corporation, Japan). Blood samples with 3.8% sodium citrate were detected the plasma coagulation (PT, APTT, TT, and FIB concentration) *in vivo* using the H1201 automatic coagulation analyzer (Jiangsu Horner Medical Instrument Co., Ltd., China).

### Plasma Coagulation Assay *In Vitro*

Rats were anesthetized with 4% pentobarbital sodium *via* IP to withdrawn blood samples from abdominal aorta and placed in a 3.8% sodium citrate vacuum tube with a blood/coagulant ratio of 9:1 and then centrifuged at 3,000 rpm for 15 min to obtain plasma. Plasma mixtures with 1.45 mL of plasma and 0.05 mL of different concentrations of PPD were incubated at 37°C for 10 min, which were used to detect PT, APTT, TT, and FIB concentration, according to the manufacturer’s protocols using the H1201 automatic coagulation analyzer.

### Human and Rat Washed Platelets Preparation

As described above, blood from healthy consented volunteers (Experimental procedures were approved by the ethics committee of the Affiliated Hospital to Changchun University of Chinese Medicine, approval no. CCZYFYLL2017-041) and male Wistar rats were collected into an anticoagulant tube of 3.8% sodium citrate, respectively. Platelet-rich plasma (PRP) was isolated as the supernatant from centrifugation at 800 rpm for 5 min. Human/rat washed platelets were prepared as before (Gao et al., 2011). PRP was centrifuged at 3,000 rpm for 5 min and washed twice with Tyrode’s buffer (137 mM NaCl, 2 mM KCl, 12 mM NaHCO_3_, 5 mM HEPES, and 0.35% BSA, pH 7.4) to obtain human/rat washed platelets.

### Platelet Aggregation Assay and ATP Release Assay

Human/rat washed platelets were adjusted to 3 × 10^8^/mL with Tyrode’s buffer including 1 mM CaCl_2_. After the incubation at 37°C for 5 min, the platelets were stimulated by various concentrations of PPD or thrombin, respectively. Platelet aggregation was performed by using a platelet aggregometer (Chrono-Log 700, Chrono-Log Co., USA) through measuring the changes in light transmission. ATP release was measured using luciferin/luciferase reagent (Chrono-lume). Additionally, vorapaxar (VP, a PAR-1 antagonist of thrombin, 10 μM), ticagrelor (TG, a P_2_Y_12_ receptor antagonist of ADP, 10 μM), and seratrodast (ST, a potent and selective thromboxane A2 receptor antagonist, 10 μM) were used to further analyze the possible mechanism of PPD on platelet activity by platelet aggregation assay.

### P-Selectin Secretion and Glycoprotein (GP) IIb/IIIa Activation on the Surface of Platelets by Flow Cytometric Analysis

Human washed platelets were incubated with different concentrations of PPD at 37°C for 5 min, and then incubated with FITC-conjugated CD62P (P-selectin marker) or FITC-conjugated PAC-1 (activated GP IIb/IIIa receptor marker) antibodies in the dark for 20 min. After stopping by adding 200 μL of phosphate-buffered saline (PBS), the samples were immediately analyzed with a BD FACSAria II flow cytometer (BD Biosciences, USA). A total of 10,000 events in triplicated from different groups were analyzed the platelet P-selectin secretion and glycoprotein IIb/IIIa activation, which was repeated at least three times to ensure the reliability.

### Determination of the Intracellular Calcium Concentration ([Ca^2+^]_i_)

As previously reported (Merritt et al., 1990), rat washed platelets were incubated with Fluo-3 AM (5 μM) at 37°C; for 60 min in the dark condition, and washed two times and suspended in Tyrode’s buffer. Platelets at the final concentration of approximately 3 × 10^8^/mL were added to the 96-well microplates (Nunc F96, Thermo Fisher Scientific, Waltham, USA) and incubated with PPD. After adding PPD, Fluo-3 fluorescence was determined at 18 s intervals for 20 min on Cell Imaging Multi-Mode Reader (Cytation 5, BioTek, Vermont, USA) with an excitation wavelength of 488 nm and an emission wavelength of 525 nm to obtain calcium kinetic curve. The [Ca^2+^]_i_ is calculated by the previous method (Tao et al., 1996) as follow: [Ca^2+^]_i_ in cytosol = 525 nM×(*F*-*F*_min_)/(*F*_max_-*F*), where 525 nM is the dissociation constant of the Fluo-3, *F* represents the fluorescence value of the sample. *F*_min_ and *F*_max_ are minimum and maximum fluorescence value amd are measured after the treatment with 10 mM EGTA and 0.1% Triton X-100, respectively.

### Measurement of cAMP, cGMP, and PAC-1

Human/rat washed platelets were incubated with methanol or PPD at 37°C for 10 min and then added the 10 mM EDTA to terminate the reaction. After freezing at -80°C and thawing at 37°C for five times, the solution was centrifuged at 3,000 rpm for 10 min at 4°C;, and the supernatant for detecting the concentrations of cAMP, cGMP, and PAC-1 using the ELISA kits according to the manufacturer’s protocol. To evaluate whether the cAMP production was involved in PPD-inhibited platelet aggregation, human washed platelets were pretreated with VP (10 μM) for 5 min and then treated with PPD to detect the cAMP level.

### Molecular Docking

In order to investigate ligand-receptor interactions between PPD and target protein of PAR1, molecular docking studies were carried out by Autodock Vina (Scripps Research Institute, La Jolla, CA, USA) (Trott and Olson, 2010). Briefly, we downloaded the three-dimensional structure of PPD (PubChem CID: 11213350) from the National Center for Biotechnology Information (NCBI) PubChem Compound database (http://www.ncbi.nlm.nih.gov/pccompound) and the crystal structure of human protease-activated receptor 1 (PAR1) bound with antagonist vorapaxar (PDB ID: 3VW7) from Protein Data Bank (http://www.rcsb.org/pdb). The PyMOL was applied for visual inspection and analysis of ligand-receptor binding mode. The binding energies (kcal/mol) were calculated to estimate ligand-binding affinity using Autodock Vina method. The best binding conformation at the active site was analyzed by covering the known key amino acid resides involved in hydrogen bond, hydrophobic interactions, and van der Waals forces.

### Statistical Analysis

Data from all experiments are presented as the mean ± standard deviations (SDs). Ordinary one-way ANOVA test (Turkey’s *post doc*) was used to analyze the differences among the groups by GraphPad Prism 8.0 software. *p* < 0.05 was considered as statistical significance.

## Results

### Effect of PPD on Bleeding Time

To detect whether PPD had the hemostatic effect, tail amputation and liver scratch models in mice were performed (Frattani et al., 2017; Yan et al., 2017). The results showed that hemocoagulase (HC) and PPD treatment significantly decreased the tail bleeding time (**Figure 1B**) and liver bleeding time of mouse (**Figure 1C**). For the tail amputation model, the tail bleeding time of mouse in 8 mg/kg of PPD group remarkably reduced compared with the NS group (*p* < 0.05, **Figure 1B**). For the liver scratch model, liver bleeding time of mouse in 4 and 8 mg/kg of PPD groups was significantly lower than that of the NS group (*p* < 0.05, **Figure 1C**). These results showed that PPD had an excellent hemostatic effect after subcutaneous injection of 4 h, which was similar with hemocoagulase.

### Effect of PPD on Routine Blood Test in Rat

**Figure 2:**
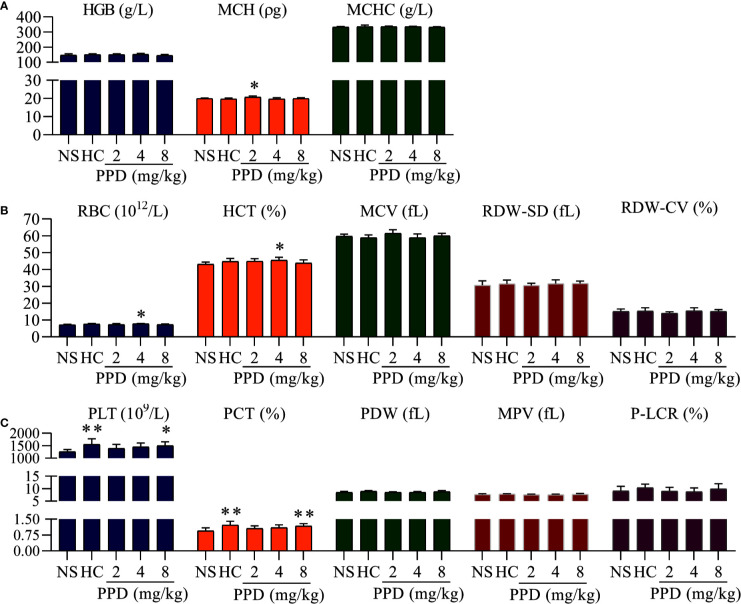
The effect of PPD on rat blood routine. **(A)** The effect of PPD on the parameters of hemoglobin, including hemoglobin counts (HGB), mean corpuscular hemoglobin (MCH), and mean corpuscular hemoglobin concentration (MCHC). **(B)** The effect of PPD on red blood cell parameters, including red blood cell (RBC) counts, hematocrit (HCT), mean corpuscular volume (MCV), red cell distribution width-standard deviation (RDW-SD), and red cell distribution width-coefficient of variation (RDW-CV). **(C)** PPD on platelet-related parameters, platelet (PLT) counts, plateletcrit (PCT), platelet distribution width (PDW), mean platelet volume (MPV), and platelet larger cell ratio (P-LCR) were measured after subcutaneously injected 4 h in rats of NS, HC, and PPD groups. The data are expressed as mean ± SD (*n* = 8). ^*^ *p* < 0.05 and ^**^ *p* < 0.01 compared to the NS group.

PPD absorbed into the blood after rat subcutaneous injection for 4 h; parameters of routine blood can directly reflect the effect of PPD on blood. As shown in **Figure 2**, the parameters of hemoglobin (HGB), red blood cell (RBC) and platelet (PLT) were significantly changed in the rat treated with PPD for 4 h; and PPD had no effect on other parameters of routine blood test, such as white blood cell counts, neutrophils, lymphocyte (Data are not shown). For the HGB parameters, mean corpuscular hemoglobin (MCH) of 2 mg/kg PPD group was increased compared with the NS group (*p* < 0.05). Other HGB parameters, such as HGB counts and mean corpuscular hemoglobin concentration (MCHC) were not changed by PPD or HC (**Figure 2A**). For the RBC parameters, compared with the NS group, RBC counts and hematocrit (HCT) increased by PPD at 4 mg/kg groups (*p* < 0.05), and other HGB parameters, such including mean corpuscular volume (MCV), red cell distribution width-SD (RDW-SD), and red cell distribution width-coefficient of variation (RDW-CV) were not influenced by PPD or HC (**Figure 2B**). Importantly, PPD at 8 mg/kg remarkably increased rat PLT counts and plateletcrit (PCT), which was similar with HC (*p* < 0.05 or *p* < 0.01, **Figure 2C**). Other platelet-related indicators, platelet distribution width (PDW), mean platelet volume (MPV) and platelet larger cell ratio (P-LCR) were not changed by PPD or HC, compared with that of the NS group (**Figure 2C**). These results showed that PPD mainly increased blood platelet count in the rats after subcutaneous injection for 4 h.

### Effect of PPD on Coagulation Parameters of Rat

**Figure 3:**
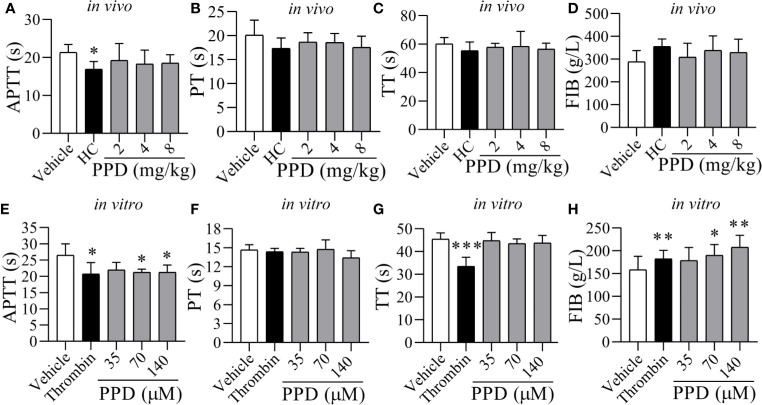
The effect of PPD on APTT, PT, TT, and FIB of rat plasma *in vivo* and *in vitro*. **(A**–**D)** Five groups of wistar male rats (Vehicle, HC, and 2, 4, and 8 mg/kg group; *n* = 8) were subcutaneously injected, respectively. After 4 h treatment, rats were anesthetized with 4% pentobarbital sodium *via* IP to withdrawn blood samples from abdominal aorta, and according to APTT, PT, TT and FIB kits’ protocol to record clotting time (s). **(E–G)** After incubation for 10 min at 37℃, 200 μL of the rat plasma mixture with PPD was blended with APTT (200 μL), PT (150 μL) or TT (150 μL) assay reagents to detect APTT, PT, or TT, respectively. The clotting times (s) was recorded immediately and monitored using an automatic coagulation analyzer. **(H)** FIB. After incubation for 10 min at 37℃, 200 μL of rat plasma mixture with PPD was blended with 100 μL thrombin assay buffer to record clotting time (s) immediately. The standard curve was drawn based on the concentration of fibrinogen (x, g/L) and clotting time (y, s) (y = -0.1505x + 57.363) for determining the content of FIB. The data are expressed as mean ± SD [**(A**–**D)** *n* = 8; **(E**–**H)** *n* = 3]. *p < 0.05, **p < 0.01, and ***p < 0.001 compared to the vehicle group.

To further determine the hemostatic effect and the mechanism of PPD, rat plasma was used for detecting coagulation parameters *in vivo* (**Figures 3A, D**) and *in vitro* (**Figures 3E, H**). The plasma coagulation *in vivo* was detected after rat subcutaneous injection for 4 h. Compared with the vehicle group, APTT, PT and TT were weakly decreased by PPD (**Figures 3A–C**); FIB was slight increased by PPD (**Figure 3D**). However, PPD had no significant differences on these 4 indicators between control and PPD-treated groups (*p* > 0.05). *In vitro* experiments, two high dose groups of PPD (70 and 140 μM) notably shortened clotting time in the APTT assay (*p* < 0.05, **Figure 3E**) compared with the vehicle group. However, there were no differences on PT and TT between the vehicle group and PPD group (**Figures 3F, G**, *p* > 0.05). Additionally, PPD significantly increased the concentration of FIB (*p* < 0.05 or *p* < 0.01, **Figure 3H**). The above results suggest that PPD could promote rat plasma coagulation, which might be related with intrinsic coagulation and FIB.

### PPD Promoted Human/Rat Washed Platelet Aggregation

**Figure 4:**
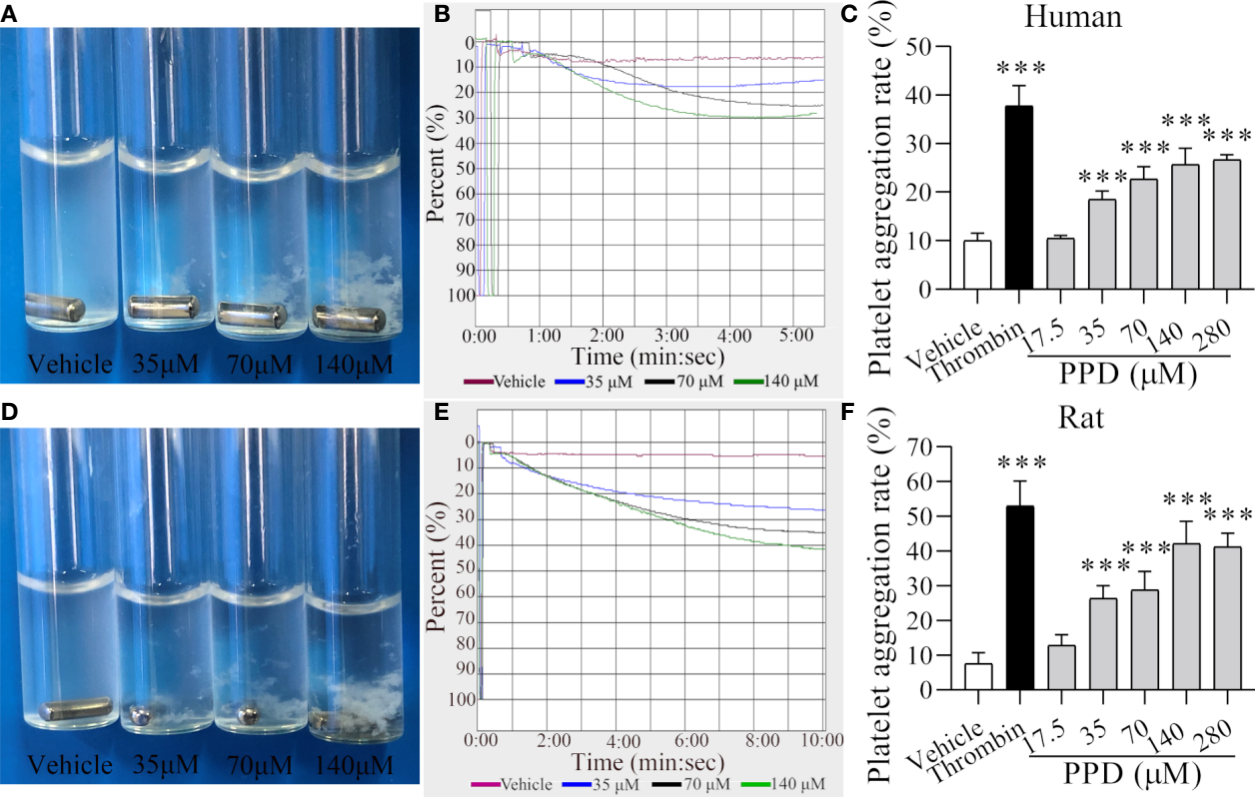
PPD induced human/rat washed platelets aggregation. **(A, D)** Human or rat platelets aggregations were observed after the stimulation with methanol or PPD (35, 70, or 140 μM). **(B, E)** Light transmission graphs of human or rat washed platelets with 1 mM Ca^2+^ after stimulation by methanol or PPD (35, 70, or 140 μM) at 37°C with shaking at 1,200 rpm/min using a platelet aggregometer. **(C, F)** Methanol, thrombin (0.5 U/mL), or different concentrations of PPD (17.5, 35, 70, 140, or 280 μM) induced the increase of human or rat washed platelet aggregation rate. After the incubation of human washed platelets (290 μL) with 1 mM Ca^2+^ for 5 min at 37°C, different concentrations of PPD (10 μL) were added and shaken at 1,200 rpm/min to detect the platelets aggregation. The data are expressed as mean ± SD (*n* = 3). ^***^ *p* < 0.001 compared to the vehicle group.

Our data indicated that PPD had shown a marked effect of hemostasis *in vivo* experiment studies mainly by affecting platelets. Therefore, we further examined the effect of PPD on platelet aggregation and analyzed its mechanism in the following experiments. After the stimulation with vehicle (methanol), thrombin, or PPD, aggregation rate of human/rat washed platelets was observed by the light transmission. As shown in **Figures 4A–C**, the human platelet aggregation rate was increased dramatically by PPD at a dose-dependent manner and attained its peak at 140 μM of PPD, and the maximal platelet aggregation rate was around 30.50% (**Figure 4C**). Similarly, PPD-induced platelet aggregation was found in rat washed platelets and shown in **Figures 4D–F**. With the increase of the concentration, PPD remarkably increased the rat platelet aggregation and arrived at its peak with the rate of about 42.17% at 140 μM (**Figure 4F**). Meanwhile, PPD induced visible platelet aggregation in aggregometer cuvettes on human and rat washed platelets (**Figures 4A, D**). These results indicate that PPD could directly induce platelet aggregation for human (maximal rate: 30.5% at 5 min) and rat (maximal rate: 42.17% at >10 min) washed platelets with a little difference.

### PPD Activated Platelet Aggregation by Promoting Calcium Influx, Releasing Granule, and Increasing PAC-1 Expression

**Figure 5:**
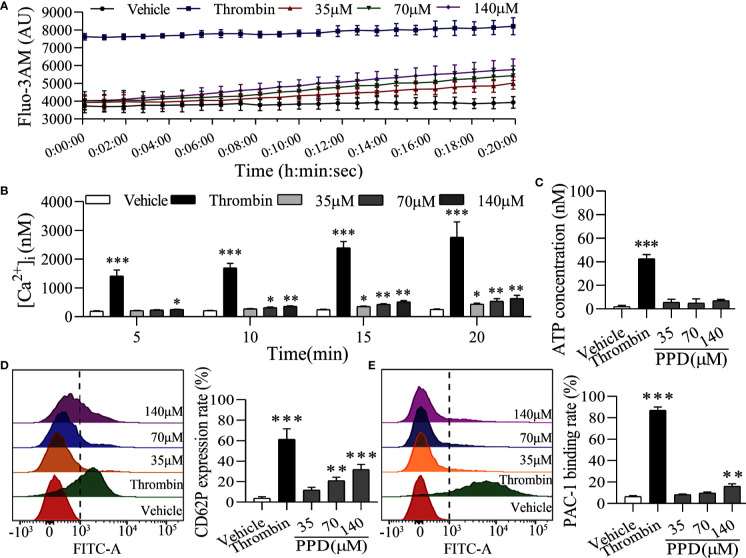
PPD activated platelets without external Ca^2+^. **(A)** The calcium kinetic curves of the human platelets stimulated by PPD or thrombin. Human washed platelets were incubated with Fluo-3 AM (5 μM) at 37°C for 60 min in the dark, washed two times and suspended in Tyrode’s buffer to obtain a final concentration of approximately 3×10^8^ platelets/mL. 140 μL of Fluo-3-loaded platelets were added to the 96-well microplates and treated with 10 μL of methanol, thrombin (0.5 U/mL) or PPD (35, 70, or 140 μM), Fluo-3 fluorescence was recorded at 18 s intervals for 20 min using Cell Imaging Multi-Mode Reader with an excitation wavelength of 488 nm and an emission wavelength of 525 nm. **(B)** The effect of methanol, PPD and thrombin on Ca^2+^ concentration ([Ca^2+^]_i_) in human platelets at different time points (5, 10, 15 or 20 min) was examined. **(C)** The effect of PPD on ATP release of human washed platelets. After incubation at 37°C for 5 min, 290 μL of platelets were added 10 μL of methanol, thrombin, or different concentrations of PPD and then incubated with 30 μL of LUME reagent (Chrono-lume) to detect ATP release using a platelet aggregometer. **(D, E)** The effect of PPD on the expression of CD62P or PAC-1 in human washed platelets. 145 μL of platelets were incubated with 5 μL of methanol, thrombin (0.5 U/mL), or PPD (35, 70, or 140 μM) at 37°C for 5 min and added into anti-CD62P or anti–PAC-1 FITC-conjugated antibodies in the dark for 20 min for flow cytometric analysis. The data are expressed as mean ± SD (*n* = 3). ^*^ *p* < 0.05, ^**^ *p* < 0.01, and ^***^ *p* < 0.001 compared to the vehicle group.

The elevation of intracellular Ca^2+^ contributes to several events of platelet activation, such as shape change, granule release, and GP IIb/IIIa activation (Gulino et al., 1992; Gilio et al., 2010). Firstly, we used Cell Imaging Multi-Mode Reader to detect the effect of PPD on the calcium kinetic curve of human platelets. As shown in **Figure 5A**, PPD moderately increased the concentration of Ca^2+^ in platelets along with the time, which was not similar with that of thrombin. Thrombin instantaneously caused a significant increase of Ca^2+^ influx into the platelets. Moreover, PPD significantly increased the concentration of Ca^2+^ in a dose- and time-dependent manner, which was less than that of thrombin (*p* < 0.05 and *p* < 0.01, **Figure 5B**). After stimulation with agonists, platelets can release granules including α-granules such as P-selection (CD62P) and dense granules such as ATP release as common markers for the quantification of platelet activation (Smyth et al., 2009). Compared with the vehicle group, we found that PPD slightly increased the ATP concentration with no significance (*p* > 0.05), which was different from thrombin (**Figure 5C**). For the CD62P expression, PPD significantly dose-dependently increased the rate of CD62P-positive platelets compared with the vehicle group, which was lower than that of thrombin (**Figure 5D**). Additionally, fibrinogen can bind its platelet receptor, GP IIb/IIIa to induce platelet aggregation. PAC-1 recognizes an epitope on the GP IIb/IIIa complex of activated platelets, which can be used for exploring platelet aggregation (Abrams et al., 1992). As shown in **Figure 5E**, the PAC-1 binding rate of human washed platelets was significantly increased by PPD treatment at 140 μM compared with the vehicle group (*p* < 0.01). The maximal rate of PPD on PAC-1 binding was 14.03%. PPD could weakly activate the platelets, but it did not directly induce platelet aggregation (data are not shown).

**Figure 6:**
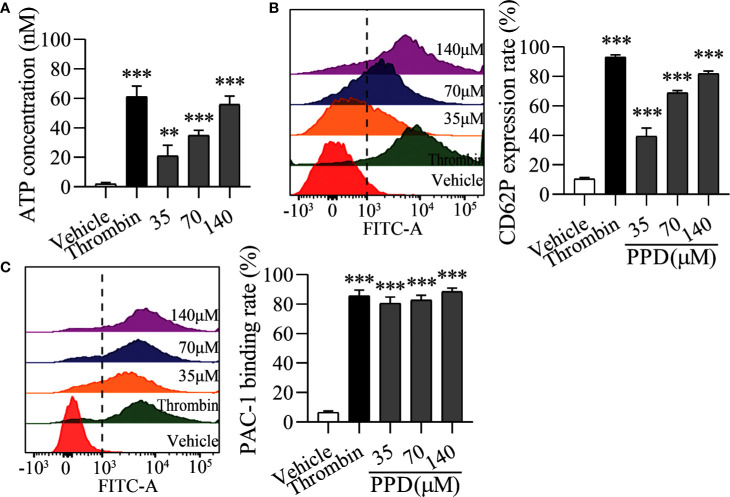
PPD activated platelets with 1 mM Ca^2+^. **(A)** After the treatment of methanol, thrombin (0.5 U/mL) or PPD (35, 70, or 140 μM) treatment and Ca^2+^ incubation, ATP release of human washed platelets was detected by 30 μL of LUME reagent (Chrono-lume) using a platelet aggregometer. **(B**, **C)** In the presence of Ca^2+^, the effect of PPD or thrombin on the rates of CD62P expression or PAC-1 binding in human washed platelets was investigated by flow cytometric analysis. The concentration of PPD or thrombin is the same as that in **(A)**. The data are expressed as mean ± SD (*n* = 3). ^**^ *p* < 0.01 and ^***^ *p* < 0.001 compared to the vehicle group.

Importantly, platelet activation stimulated by various agonists is strongly dependent on the increased Ca^2+^ concentration in cytoplasm (Davlouros et al., 2016). Therefore, we further observed the effect of PPD on platelet activation in the Ca^2+^-dependence. As shown in **Figure 6A**, PPD dose-dependently caused a significant increase of ATP release of human washed platelets incubated with 1 mM Ca^2+^, compared with the vehicle group (*p* < 0.001), which was stronger than that of platelets without Ca^2+^ incubation (**Figure 5C**). After addition with 1 mM Ca^2+^, CD62P expression rate of PPD group was remarkably increased compared with the vehicle group, which reached to 81.90% at 140 μM (*p* < 0.001, **Figure 6B**). PPD-induced CD62P expression in platelets under stimulation with Ca^2+^ was higher than that in the absence of Ca^2+^ (**Figure 5D**). After PPD treated and Ca^2+^ incubation, the PAC-1 binding rate of human washed platelets was significantly increased and arrived to 81.14% at 140 μM, which was dose-dependent and similar as that of thrombin (**Figure 6C**). Similarly, the effect of PPD on PAC-1 expression was dependent on Ca^2+^ concentration. The data above demonstrated that PPD obviously increased ATP release and the levels of CD62P and PAC-1, which was mediated by the amount of Ca^2+^. Overall, PPD treatment markedly promoted platelet activation by promoting calcium influx, releasing granule and increasing PAC-1 expression.

### PAR-1 Signaling Involved in PPD-Induced Platelet Activation, Release, and Aggregation

**Figure 7:**
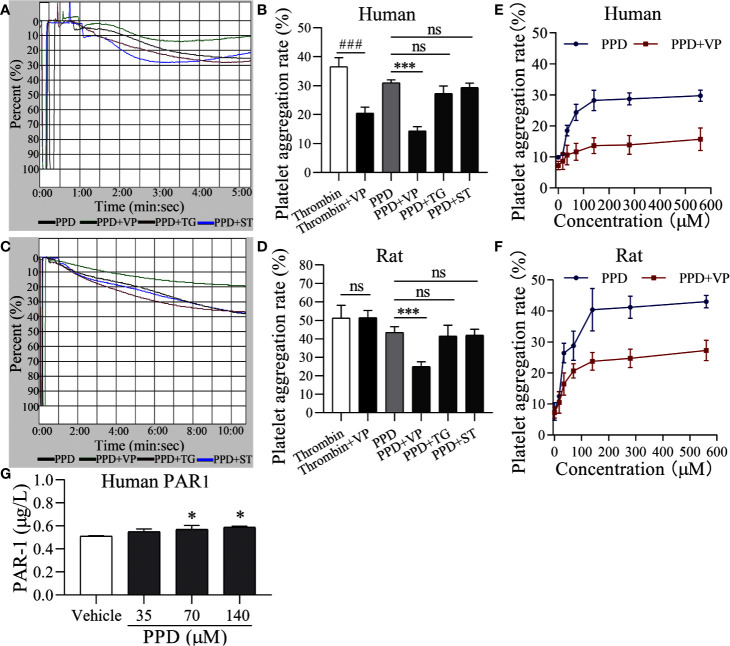
PAR-1 signaling involve in PPD-induced platelet aggregation. **(A**, **C)** Light transmission graphs of PPD (70 μM) combined with VP (10 μM), TG (10 μM), or ST (10 μM) on human/rat washed platelets. **(B**, **D)** The aggregation rate in human/rat platelets of PPD (70 μM) alone or combined with VP (10 μM), TG (10 μM), or ST (10 μM). **(E**, **F)** VP inhibited increased human/rat washed platelet aggregation by PPD at different concentrations (17.5, 35, 70, 140, 280, and 560 μM). **(G)** PPD increased the expression of PAR1 on platelet surface. Human washed platelets were incubated with methanol and PPD (35, 70, and 140 μM) at 37°C for 10 min and then added the 10 mM EDTA to terminate the reaction. After freezing at -80°C and thawing at 37°C for five times, the solution was centrifuged at 3,000 rpm for 10 min at 4°C to obtain the supernatants for detecting the concentration of PAR1 using the ELISA kits. The data are expressed as mean ± SD (*n* = 3). ns was no significant between the two groups. ^###^ *p* < 0.001 compared to the thrombin group and ***p < 0.001 compared to the PPD group in panel **B** and **D**; *p < 0.05 compared to the Vehicle group in panel **G**.

To further identify the molecular mechanism of PPD on platelet activation, we used three inhibitors, such as VP, TG, and ST for PAR-1, P2Y_12_, and thromboxane A2 receptor, respectively. As shown in **Figure 7A**, PPD-induced human platelet aggregation was markedly inhibited by VP (*p* < 0.001), but TG and ST had no effect on PPD-induced platelet aggregation (**Figures 7A, B**). Similar results were found in the PPD-induced rat platelet aggregation (**Figures 7C, D**). We further investigate the effect of different concentrations of PPD combined with VP on platelet aggregation. The results in **Figures 7E**, F showed that the rates of human and rat platelet aggregation were gradually increased by PPD. The combination of PPD with VP notably reduced the aggregation rates in human and rat platelets (**Figures 7E, F**). These findings showed that PPD (70 μM)-induced human/rat platelet aggregation might be mediated by regulating PAR-1. Therefore, the expression of PAR1 was detected on PPD-induced human platelets. The **Figure 7G** showed that PPD significantly increased the expression of PAR1 on platelet surface (*p* < 0.05).

**Figure 8:**
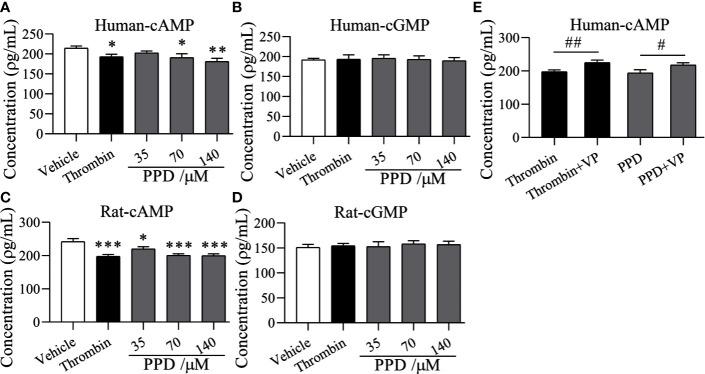
The effect of PPD on cAMP and cGMP levels in human and rat platelets. **(A**, **B)** The effect of PPD on cAMP or cGMP concentration in human washed platelets. **(C**, **D)** The effect of PPD on the level of cAMP or cGMP concentration of rat washed platelets. **(A–D)** Human/rat washed platelets were incubated with methanol, thrombin (0.5 U/mL), and PPD (35, 70, and 140 μM) at 37°C for 10 min and then added the 10 mM EDTA to terminate the reaction. After freezing at -80°C and thawing at 37°C; for five times, the solution was centrifuged at 3,000 rpm for 10 min at 4°C to obtain the supernatants for detecting the concentration of cAMP or cGMP using the ELISA kits. **(E)** VP reversed the decrease of the cAMP content caused by PPD in human platelets. Human washed platelets were pretreated with VP (10 μM) for 5 min and then treated with PPD for measuring cAMP level as the procedure described above. The data are expressed as mean ± SD (*n* = 3). ns was no significant between the two groups. ^*^ *p* < 0.05, ^**^ *p* < 0.01, and ^***^ *p* < 0.001 compared to the vehicle group, ^#^ *p* < 0.05 and ^##^ *p* < 0.01 compared to the two groups without and with VP.

**Figure 9:**
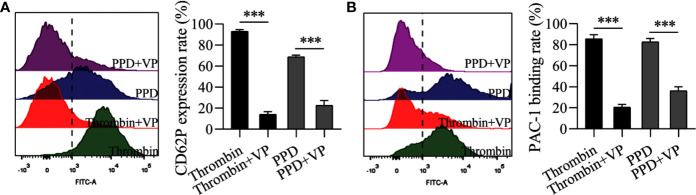
VP inhibited the rates of CD62P expression and PAC-1 binding of human washed platelets induced by PPD or thrombin. **(A)** VP inhibited PPD- or thrombin-induced the CD62P expression rate in human platelets with 1 mM Ca^2+^. **(B)** VP inhibited the PAC-1 binding rate of PPD- or thrombin-induced human washed platelets with 1 mM Ca^2+^. **(A, B)** Human washed platelets (145 μL) with 10 μL VP (10 μM) were incubated with 5 μL of thrombin (0.5 U/mL) or PPD (140 μM) at 37°C for 5 min and then added into anti-CD62P or anti–PAC-1 FITC-conjugated in the dark for 20 min for fluorescence analysis. The data are expressed as mean ± SD (*n* = 3). ^***^ *p* < 0.001 compared to the two groups without and with VP.

Importantly, PAR-1 is coupled to G_q_ and G_i_ proteins that lead to a reduction in cAMP (Yang et al., 2002), and cGMP concentration is increased by thrombin-induced platelets (Kobsar et al., 2012); therefore, we detected the effect of PPD on the levels of cAMP and cGMP in human/rat washed platelets. As shown in **Figures 8A, B**, PPD inhibited cAMP production of human platelets in a dose-dependent manner compared with the vehicle group (*p* < 0.05 or *p* < 0.01), which had no effect on cGMP production in human platelets. Similar findings for cAMP and cGMP concentrations by PPD were found in rat platelets (*p* < 0.05 or *p* < 0.001, **Figures 8C, D**). The combination of PPD with VP reversed PPD-induced the reduction of cAMP concentration in human platelets by PPD (*p* < 0.05, **Figure 8E**). Meanwhile, we investigated the combination of PPD with VP on CD62P expression and PAC-1 binding in human washed platelets. The results found that PPD-induced the increases of CD62P and PAC-1 were markedly inhibited by VP (**Figure 9A**, *p* < 0.001 and **Figure 9B**, *p* < 0.001). The effect of PPD on platelet activation was similar with that of thrombin. The above results showed that PPD induced platelet activation, release and aggregation through regulating PAR-1 pathway.

### Molecular Docking Between PPD and PAR1

**Figure 10:**
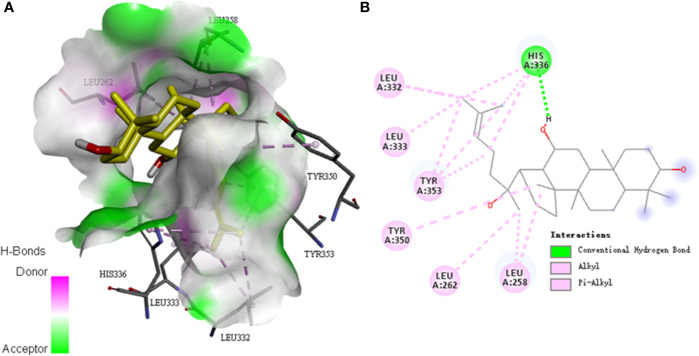
Molecular docking between PPD and PAR1. **(A)** The model of PPD (PubChem CID: 11213350) binding with the crystal structure of human PAR1 bound with antagonist vorapaxar (PDB ID: 3VW7) by using the Autodock Vina. Docking score = -6.6 kcal/mol^−1^. Pink, H-bonds donor; green, H-bonds acceptor. **(B)** Two-dimensional interaction map of PPD and the crystal structure of human PAR1 bound with antagonist vorapaxar. The dotted lines indicate potential interactions between amino acid residues and PPD. Green dotted line, conventional hydrogen bond; pink dotted lines, alkyl and pi-alkyl.

The docking study was carried out to find the best binding pose of PPD with PAR1 based on published monomeric crystal structure of human PAR1 bound with antagonist vorapaxar (PDB ID: 3VW7) using the Autodock Vina. We docked PPD to crystal structure of human PAR1 bound with antagonist vorapaxar, the results showed a promising docking score with -6.6 kcal/mol. The hydrophobic part of PPD, the scaffold of triterpene, extended deeply into the pocket and interacted with hydrophobic residues, such as Leu258, Leu262, Leu332, Leu333, His336, Tyr350, and Tyr353 (**Figure 10**). Meanwhile, the hydrogen bond formed between the hydroxyl at the C-12 position of triterpene with His336 (**Figure 10**), which enhanced the binding affinity of PPD to the PAR1. Collectively, PPD can bind and target PAR1.

## Discussion

*P. notoginseng* is a highly valued Chinese materia medica with a hemostatic effect and mainly used for the treatment of different bleeding scenarios, including traumatic bleeding, hematemesis (vomiting of blood), lower gastrointestinal bleeding, snakebite, abnormal menstrual cycle, lochia, and infection-meditated corneal ulcers (Yang et al., 2018). The modern medical research found that dencichine and notoginsenoside Ft1 showed the hemostatic effect (Huang et al., 2014; Liu et al., 2019). In the present study, we evaluated the hemostatic effects of PPD in *P. notoginseng* on human/rat platelets and coagulation parameters.

Many factors influence the bleeding time, such as the capillary function, tissue shrinkage ability, count and function of cell factor and platelets, function of the fibrinolytic system of mice (Jalaer et al., 2017). First, we detected that PPD stopped bleeding time at mouse tail amputation and liver scratch models *in vivo*. The results showed that PPD had a good hemostatic effect on bleeding models of the mouse (**Figure 1**). Second, the effect of PPD on rat routine blood test was evaluated. The results showed that PPD increased remarkably RBC counts, HCT, PLT counts, PCT and MCH of rat serum (**Figure 2**). Lin et al. reported that PPD significantly increased the peripheral white blood cell count, bone marrow cell count in CTX-treated tumor-bearing mice (Lin et al., 2013). RBC counts and PLT counts were increased by PPD, which indicated that PPD could promote the hematopoiesis and thrombopoiesis (Yang et al., 2009) after rat subcutaneous injection 4 h. HCT and related hematologic variables such as HGB and RBC counts are closely related to thrombogenesis in a general population (Braekkan et al., 2010). The increase of RBC counts and PLT counts participated in the hemostatic process to accelerate thrombosis at the site of bleeding with the PPD treatment group. Activated platelets secrete an abundance of granules to maintain and amplify the initial response of the platelets and stimulate more circulating platelets that are recruited to aggregates at the injured vessel wall (Blair and Flaumenhaft, 2009). And RBC is compressed to close-packed polyhedral structures with platelets and fibrin on the surface in contracted clots and thrombi (Cines et al., 2014). The variation of the indexes as mentioned above indicated that PPD could accelerate the hemostasis on blood vessel or other tissue wounds *in vivo*.

To further confirm the coagulation pathways of PPD, coagulation parameters were carried out *in vivo* and *in vitro*. APTT and PT are sensitive and commonly used screening test for intrinsic and extrinsic coagulation system, respectively (Lippi et al., 2009). APTT reﬂects the level of coagulation factor VIII, IX, X, XI, and XII in plasma; PT reflects the overall activity of coagulation factor III, VII, V, and X in plasma (Cowan et al., 1981; Lippi et al., 2009); TT primarily reflects whether there is an abnormal level of fibrinogen, anticoagulant, and fibrinolytic substance in the common pathway of coagulation process that fibrinogen converted to fibrin (Li et al., 2008). At the end of these processes, fibrinogen is transformed into fibrin, which transformed the blood from the collosol state to the gel state (Jaeger and Labarrere, 2003). In this study, *in vivo* experiment studies demonstrated that PPD weakly increased APTT, PT, and FIB (**Figure 3**, *p* > 0.05); *in vitro* experiment, the decrease of APTT and the increase of FIB content suggested that PPD had good hemostatic effects by activating endogenous coagulation system to accelerate the formation of FIB. These experiment results demonstrated that PPD had hemostatic effects; so we further examined the effect of PPD on platelet aggregation and explored its mechanism. Activated platelets release dense granules (such as 5-HT, ADP, ATP, histamine, CD63, etc.) and a-granules (P-selectin, PF4, vWF, and thrombospondin-1, etc.) (Blair and Flaumenhaft, 2009), which modulate the function of interacting platelets and blood vascular cells. Some granules such as ATP, ADP, 5-HT, vWF stimulate additional circulating platelets that are recruited to form the aggregates. Our results had shown that PPD activated the platelets, and increased ATP content and CD62P release of human platelets to induce platelet aggregation. Additionally, GP IIb/IIIa is exposed on platelets surface to enable the binding of soluble ligands and activation-dependent changes in the conformation of GP IIb/IIIa can be detected by specific antibody (PAC-1) (Jurk and Kehrel, 2005). PPD increased the PAC-1 binding rate of activated platelets, which promoted fibrinogen into insoluble fibrin and participated in the hemostatic process.

Human platelets maintain a low resting [Ca^2+^]_i_ estimated to be around 50–100 nM (Vicari et al., 1994). An increase in platelets [Ca^2+^]_i_ is a pivotal signaling event during platelet activation. PPD markedly increased [Ca^2+^]_i_ of rat platelets after 5 min of incubation, which was slower and more extended time than thrombin (**Figure 5A**). Ca^2+^ signaling participated in the regulation of platelet activation, shape change, granule release, thrombus formation, and GP IIb/IIIa activation (Gulino et al., 1992; Gilio et al., 2010). In the absence of Ca^2+^, PPD promoted the GP IIb/IIIa activation (PAC-1 binding rate) and CD62P expression, which was very weaker than that of thrombin (**Figure 5**). When 1 mM Ca^2+^ was added to platelets, PAC-1 binding rate, CD62P expression and ATP release of platelets were noticeable rises treated with PPD (**Figure 6**). These results showed that the high concentration of Ca^2+^ enhanced the activation of PPD on platelets and collectively accelerated platelet aggregation (**Figure 4**). In other words, PPD induced the platelet aggregation that might be dependent on the high concentration of Ca^2+^.

The platelet membrane has many receptors, such as GPIb/V/IX, GPVI, a2β1, PARs, P2Y_1_, P2Y_12_, thromboxane A2 receptor (TP), and integrins (Jurk and Kehrel, 2005). Three inhibitors, VP, TG, and ST activate platelets by binding PAR-1, P2Y12, and TP, respectively, which were used to screen the potential sites of PPD on platelets (Wouters et al., 1999; Husted and van Giezen, 2009; Judge et al., 2015). The results revealed that only VP could inhibit PPD-induced platelet aggregation (**Figure 7**). PAR1 and PAR4 were demonstrated to mediate most of platelet responses to thrombin (Kahn et al., 1999). PAR1 has a higher affinity for thrombin than PAR4, and its activation results in a faster and stronger Ca^2+^ increase. PAR1 couples G12/13, Gq, and Gi/z families of heterotrimeric G proteins, thereby connecting coagulation to a host of intracellular signaling pathways (Coughlin, 2005). The α-subunits of G12/13 bind RhoGEFs (guanine-nucleotide exchange factors, which activate small G proteins such as Rho), providing a pathway to Rho-dependent cytoskeletal responses that are likely to be involved in shape changes in platelets (Klages et al., 1999). Gα_q_ activates phospholipase Cβ (PLCβ), triggering phosphoinositide hydrolysis, which results in calcium mobilization (Wang et al., 2002), and Gα_i_ reduces the level of cAMP in platelets (Yang et al., 2002). In molecular docking study, PPD interacted with many residues of crystal structure of human PAR1 bound with antagonist vorapaxar in the pocket, which was further determined the possible bonding points between PPD and PAR1 (**Figure 10**). At same time, the **Figure 7G** showed that PPD significantly increased the expression of PAR1 (*p* < 0.05). These results demonstrated PPD had similar interaction site of platelets as thrombin and might bind to PAR1 in platelets to promote platelet aggregation, which was inhibited by VP (**Figure 7**). Moreover, the reduced level of cAMP in platelets induced by PPD was returned to normal level after adding VP (**Figure 8E**). In addition, VP reversed the results that PPD stimulated platelets to release dense granules (P-selection) and increase PAC-1 binding rate (**Figure 9**). All of the above results showed that PPD induced platelet aggregation that mainly was involved in PAR1 pathway.

In conclusion, PPD, an aglycone of protopanaxadiol-type ginsenosides, directly induced platelet aggregation and promoted blood hemostasis. PAR1 pathways played an essential role in mediating the hemostatic effect of PPD, which was dependent on calcium signaling. PPD is a critical ingredient of *P. notoginseng* for hemostatic effect, which might act as hemostatic medicine for clinical therapy of hemorrhage.

## Data Availability Statement

The raw data supporting the conclusions of this article will be made available by the authors, without undue reservation.

## Ethics Statement

The animal study was reviewed and approved by Bioethics Committee of Changchun University of Chinese Medicine and the Institutional Animal Care (approval no. 20190133).

## Author Contributions

Writing—original draft: HZ and DP. Methodology: XW, WS, and XT. Conceptualization: DZ, LS, and BS. Writing—review and editing: XB and XL.

## Funding

This work was supported by the National Key Research and Development Program of China (nos. 2017YFC1702103 and 2017YFC1702106), the National Natural Science Foundation of China (no. U19A2013), the Science and Technology projects of Education Department of Jilin Province (no. JJKH20200910KJ), the Science and Technology Development Plan Project of Jilin Province (nos. 20190101010JH and 20190304095YY), and Cultivation Fund Project of Changchun University of Chinese Medicine.

## Conflict of Interest

The authors declare that the research was conducted in the absence of any commercial or financial relationships that could be construed as a potential conflict of interest.
